# Clarifying learning experiences in student-run clinics: a qualitative study

**DOI:** 10.1186/s12909-018-1352-6

**Published:** 2018-10-26

**Authors:** Tim Schutte, Jelle Tichelaar, Erik Donker, Milan C. Richir, Michiel Westerman, Michiel A. van Agtmael

**Affiliations:** 1 Department of Internal Medicine, pharmacotherapy section, Amsterdam UMC, Vrije Universiteit Amsterdam, Amsterdam, The Netherlands; 2 RECIPE (Research & Expertise Center In Pharmacotherapy Education), Amsterdam, The Netherlands; 3 Department of Internal Medicine, Amsterdam UMC, Vrije Universiteit Amsterdam, Amsterdam, The Netherlands; 4 Research in Education, VUmc School of Medical Sciences, Amsterdam UMC, Vrije Universiteit Amsterdam, Amsterdam, The Netherlands; 5 Department of Internal Medicine, Zaans Medisch Centrum, Zaandam, The Netherlands

**Keywords:** Workplace based learning, Student-run clinics, Qualitative research

## Abstract

**Background:**

Student-run clinics (SRCs) are outpatient clinics run and organized by undergraduate medical students. While these clinics offer participating students multiple learning opportunities, little is known about how participation in an SRC contributes to learning and how this learning is influenced.

**Methods:**

In this qualitative clarification study, we conducted semi-structured interviews with a purposive sample of 20 students and student-coordinators participating in our learner-centred SRC (LC-SRC), to gain in-depth insight into their experiences and learning. These interviews were analysed using Glaser’s approach to grounded theory.

**Results:**

Analysis revealed that responsibility, authenticity, and collaboration described how SRC participation contribute to learning. Responsibility encompassed the responsibility students had for their patients and the responsibility that the student coordinators had for the students. Authenticity reflected the context and tasks in the LC-SRC. Collaboration covered collaboration with other students, with student coordinators, and with clinical supervisors. These three themes are interrelated, and together enhanced motivation and promoted patient-centred learning in both the LC-SRC and the regular curriculum.

**Conclusions:**

Learning in an LC-SRC is highly dependent on students’ feelings of responsibility for real authentic tasks and is stimulated by extensive collaboration with fellow students and supervising doctors.

**Electronic supplementary material:**

The online version of this article (10.1186/s12909-018-1352-6) contains supplementary material, which is available to authorized users.

## Background

Student-run clinics (SRCs) are (free) outpatient clinics for underserved and uninsured patients, run and organized by undergraduate medical and paramedical students. While these clinics offer students multiple learning opportunities [1–5], little is known about the actual effects of participation on students’ learning outcomes with regard to knowledge, skills, and behaviour [1]. Moreover, there has been little qualitative analysis how participation in an SRC contributes to learning and how this learning is influenced. Such analysis is essential to establish how SRC participation contributes to medical students’ development of skills and competencies, especially if the SRC concept is to be incorporated into medical education.

Previous (qualitative) studies have established the popularity of SRCs among students and described the opportunities and experiences that can be gained from SRC participation [5–9]. Chen et al. recently emphasized the educational value of early undergraduate clinical experience obtained in SRCs, which they consider a result of engagement [10]. However, not every (early) experience gives rise to student engagement and learning [11, 12]. Although sitting next to a clinical supervisor during a consultation may count as experience, it could be postulated that the engagement and effect on learning is minimal [13].

Despite the apparent learning opportunities for undergraduate medical students participating in SRCs, two key questions remain to be answered: *‘How does it work’ and ‘why does it work’*. Studies answering these types of questions are described by Cook et al. as clarification studies [14]. To better understand the learning experiences in a SRC, our research questions were:

1. How does SRC participation contribute to learning?
2. Which factors are influential to learning in a SRC?

## Methods

### Setting

While SRCs were originally for underserved and uninsured patient populations [1, 4], most patients in many European countries have some form of medical insurance, so there is relatively little need for SRCs [1, 15]. This made it necessary to adapt the original SRC concept to accommodate insured patients, but with a focus on medical education [15]. In 2012 we founded the first European SRC focused on medical education with insured patients in the VU University Medical Center [15]. This new learner-centred SRC (LC-SRC; “Studentenpoli” in Dutch) was set up with a view to providing medical students with clinical learning opportunities, such as patient consultations, patient management, and pharmacotherapy [15]. Participation in this LC-SRC is an extracurricular activity, available to all medical students following their training in the VU University Medical Center School of Medical Sciences. The medical curriculum consists of 3 years of preclinical education (BSc), followed by 3 years of clinical education (MSc/MD degree). The preclinical education includes two work placements – a first-year placement in a nursing team (either hospital/nursing home; full-time for 1 month), and a second-year placement in general practice (5 half days over a semester).

As the results of a pilot and feasibility study of the LC-SRC were encouraging [15], the project was scaled-up and now includes subprojects focused on thyroid-diseases/endocrinology, adverse drug reactions, cardiovascular risk management, polypharmacy, and general internal medicine, in addition to the above-mentioned patient consultations, patient management, and pharmacotherapy [16–18].

In the LC-SRC, before students carry out a patient consultation they are sent a schedule together with instructions (what to expect, what to study) by a student coordinator, who is usually a third-year medical student. Student participants then prepare for consultations in teams of 2–3 students, by reading the patient’s electronic medical record, practising aspects of the physical examination, and drawing up a concept (treatment) plan for the consultation. They are assisted by the student coordinator, who coaches the students and gives constructive feedback. Before and/or half-way through each consultation, student participants consult their clinical supervisor (general practitioner or consultant in internal medicine) and present their treatment plan for final approval. After the patient consultation, students receive feedback from their student coordinators, clinical supervisors, and patients (by means of a patient feedback form).

### Design

This qualitative study made use of semi-structured interviews to gain in-depth insight into the experiences and learning of students participating in our LC-SRC. We considered Glaser’s approach of the grounded theory the most suitable design to study our research questions from a clarification study point of view [14, 19–21]. This approach means looking at data with an open-mind, without a prior theoretical framework, and focusing on emerging themes, concepts, and categories [19, 21]. The Consolidated criteria for Reporting Qualitative studies (COREQ) were used to design the study [22].

### Interview development

The interview items were developed on the basis of the research questions, previous experience, and a search of the literature on undergraduate medical education and early clinical experiences. The topics covered students’ motivation for enrolment, their experience with participation, their perception of the interaction with other students and supervisors, their views on possible differences between the LC-SRC and the regular curriculum, and facilitators and barriers to learning and participating in the LC-SRC [see “Additional file 1” for the topic list]. Slight changes were made to some of the initial questions on the basis of two pilot interviews and a list of probes was added to the interview guide.

### Participants and procedure

Students who frequently (> 10 times) took part in the LC-SRC either as participant or as coordinator were eligible for this study. This purposive sampling enabled us to gain an overview of learning in an LC-SRC from the perspective of both participant and coordinator. The students were interviewed between July and December 2016 by either T.S. or E.D. (in presence of T.S.), and no others were present besides the participant and researcher(s). We approached 39 students by e-mail, of whom 21 consented to participate in the study. Reasons for not participating were mainly organizational (e.g. students were busy during clerkships in other hospitals or with other activities). The interviews took place at the VU Medical Center and were scheduled by e-mail. All interviews were recorded after participants’ consent and took about 60 min, as established in two pilot interviews. We continued data collection until theoretical saturation was reached. No repeat interviews were conducted.

The ethics review board of the Netherlands Association for Medical Education (NVMO) approved the research proposal (ID 2016/738). All participants were informed about the study in advance, gave their written consent, and participated on a voluntary basis. All interviews were numbered and stored separately from the name of the participant, the date of interview, and other information that could identify the participant.

### Analysis

All interviews were transcribed verbatim by T.S. and E.D., and the complete transcripts were e-mailed to the interviewees as respondent validation (member check). After adjustments were made in response to any comments they might have had and the interviewees had given their consent, we imported the transcripts into the qualitative data analysis program MAXQDA (version 12; Marburg, Germany). Interview transcripts and field notes were analysed as soon as possible after interview completion, to enable the iterative process and exploration of emerging themes. After completion of the open coding of nine interviews (T.S.), two other researchers (M.W. and J.T.) each recoded two transcripts using the (preliminary) codes of the first coding round. Coding differences were discussed until full consensus on the coding system was achieved. In the second level of analysis, we continuously compared the codes, their meaning and their interrelationships so as to form comprehensive categories and themes. The research team discussed the results until full consensus was achieved after four meetings.

## Results

From July to December 2016, we interviewed 20 students, after which saturation was reached. These students were 6 current participants, 7 previous participants, and 7 coordinators (see Table 1 for participant characteristics). Analysis of the interviews for these three groups revealed that the same themes described how SRC participation appeared to contribute to learning, namely, responsibility, authenticity, and collaboration. These three themes are interrelated. Two subthemes of responsibility emerged, namely responsibility for (1) patients, and (2) students, and three subthemes of collaboration emerged, namely, with (1) students, (2) student coordinators, and (3) clinical supervisors.

**Table 1:** Participant characteristics

	Participants (*n* = 6)	Student coordinators (*n* = 7)	Previous participants (*n* = 7)
Sex			
Male	2 (33.3%)	2 (33.3%)	2 (28.6%)
Female	4 (66.7%)	4 (66.7%)	5 (71.4%)
Study year			
Range	2nd – 4th year	2nd – 4th	4th – 6th year
Median	2nd year	3rd year	4th year
Mean	2.5 year	3.0 year	4.9 year
Age			
Range	18–23 years	19–22 years	22–28 years
Median	21 years	20 years	24 years
Mean	20.7 years	20.4 years	24.0 years

### Responsibility

The theme responsibility encompassed the responsibility students had for their patients and the responsibility that the student coordinators had for the student participants and how this related to learning. Because they were responsible for patient care, the student participants worked hard to prepare for their consultations, motivated by both curiosity and their desire to be optimally prepared. Likewise, the student coordinators felt responsible for, and were stimulated by, the participating students. They described learning subject matter skills, leadership, educational, and organization competencies and to have gained insight in the organizational aspects of the healthcare system.

### Responsibility for patients

Participation in the SRC meant that students, working as a team, would carry out a patient consultation. Students found this a new and stimulating challenge as it carried responsibility, which is lacking in the regular medical curriculum. However, this responsibility was a burden to some students, costing them time and energy at the expense of their regular curriculum. The responsibility the students felt towards their patients meant that they worked hard to prepare as best they could for the consultation. The consultation involved the student participants interacting with patients and clinical supervisors, which was a new experience. However, they felt that they were trusted and taken seriously by both. Even so, this responsibility created anxiety about missing something important.

*“Because you are working with real patients, you feel responsible for coming up with the right diagnosis, for giving the correct information. You cannot miss anything”. (Interview 20 (I-20)).*

Working in a team with fellow students and student coordinators provided student participants with support and helped diminish their anxiety. While reflecting on their experiences, student participants described responsibility as an important driver for their learning and development.

*“Here [in the LC-SRC] it is more like, you are more engaged. I was asking myself questions, how come, and why. This is really different compared to learning in the regular curriculum. For instance, I would be only learning the core symptoms of Parkinson’s disease, not thinking so much about whether she [the patient] is using other drugs, how does it work, and how would this affect her symptoms. So, maybe I am a bit lazy when I am studying for my exams, since I am only learning what I need to learn in order to pass. Maybe that’s my fault. Conversely, in the LC-SRC you are really stimulated because of the feedback and the responsibility, because you are really working on someone’s health problem. Yeah, that is what I really liked.” (I-19).*

### Responsibility for students

When former student participants were asked to join the student-coordinator team, they were enthusiastic and considered it a great honour. They expected to have new learning opportunities but also less direct patient contact. They thought that they might experience difficulties in the transition to their new role and responsibilities, which puts them between the students and clinical supervisors.

*“As a coordinator, I think it is important to be able to recognize unprofessional behaviour, and in this coordinating role you have to discuss this and handle it accordingly. In the end, the coordinator is meant to assist the student, but you are also responsible for the LC-SRC, and therefore professional behaviour is a requirement. This is a major responsibility, so if I think a student does something wrong, this has to be mentioned.” (I-07).*

The role and responsibilities of the student coordinators in preparing for a consultation seemed to depend on their experience. Their activities ranged from practising the entire consultation and explaining all details of the electronic medical record together with various logistic issues to only being available for questions. The student coordinators felt responsible for the definitive plans the students proposed to the clinical supervisors, and therefore wanted to guide and advise them correctly. The coordinators helped each other to acquire the necessary coaching competencies and also to learn about the topics or subject matter that the student participants would encounter.

### Authenticity

Authenticity entails the context and tasks that student participants encounter when working in the LC-SRC, which reflect those of their future workplace. The authenticity of the LC-SRC meant that students experienced real clinical practice, and expressed to have learned what is important in the care of real patients. They found this highly motivating and patient centred.

*“Just learning from books becomes boring at a time. And then you think, why do I do this to myself? But then, when you are having your consultations with the LC-SRC, you will realise, this is why; It is so nice.” (I-18).*

Students’ enthusiasm for LC-SRC participation was based on a combination of expectations regarding early clinical experiences and additional learning opportunities, including applying knowledge in practice, especially regarding pharmacotherapy. This opportunity to experience the future workplace was the main reason why some students joined the LC-SRC.

*“I was really in doubt whether I should continue [with my medical study]; however, I wanted to be sure before I decided. Therefore, I thought participating in the project would offer me a true view of practice.” (I-09).*

*“[I wanted to participate] because I was wondering how the hospital works. The first years of our study we are overwhelmed with theories and pathophysiology, but I was so curious how real practice would be..” (I-01).*

This authentic situation required students to perform an actual consultation with real patients and to prepare for this, by studying and practising skills regarding specific diseases, diagnostics, and (pharmaco) therapy. Preparing for a consultation was considered educational and useful, as were the general competences and skills needed for this, such as the ability to use the electronic patient record, to take and summarize a medical history, and to concisely discuss findings with the clinical supervisors. Students even discussed intimate subjects with patients during the consultations, which they experienced as special. This authentic interaction also included the uncertainty of clinical practice.

*“A consultation can go differently than expected. For example, when a patient has new symptoms or reacts unexpectedly. For instance, a patient can disagree with the plan you propose. Or patients can ask lots of additional questions, also questions for which you have no answers prepared. Therefore, and certainly for medication, it is very important to know some things by heart, and above all to be able to explain it to your patients. Therefore, I think it’s very educational.” (I-02).*

In the patient feedback to the student participants (via feedback forms), patients expressed satisfaction and provided constructive feedback that was perceived as being exceptionally useful. Reflecting on their LC-SRC experiences, students thought the authentic setting helped them to understand the relation between symptoms, diseases, and treatments. This was very didactic and motivating, especially seeing the results of your own actions.

*“Stopping medication appears quite simple, still it gives you a feeling of being very helpful. Because without these consultations, one lady would have never stopped with two medicines, and another man would not have stopped smoking. Thereby you feel good, you have the feeling of being useful.” (I-03).*

Both learning about networks of symptoms, diseases, and treatments and longitudinal learning were new (patient-centred) learning strategies for the student participants. They felt that this type of learning would be effective when applied to future patients. Some students reported having used the learning methods, skills, and knowledge acquired in the LC-SRC to the regular medical curriculum.

*“I tried to transfer how I learned in the LC-SRC to my regular learning, including not taking everything for granted, continuously asking myself question like “how?” and “why?”, foregrounding everything,compared to learning just facts as I used to do. […] This has really benefited me a lot. I mean it is the reason I am now a nominal student without re-examinations. That was really beyond my expectations [given the participant’s specific situation] […]. I have really learned how to learn.” (I-04).*

Furthermore, the student participants saw the LC-SRC as authentic and completely different from their regular curriculum. They considered their regular curriculum, with simulation, cases, and work placements, as not always being authentic (fake, not role of medical doctor, and exaggerated) with too few patient encounters.

*“The regular curriculum […] for me it is kind of fake. For instance there is an actor that is supposed to act thwart [..] it is kind of useless and overdone. In real practice there are also such patients, but then you will handle it more naturally [...]. Such simulations with an actor does not really stay with you, as opposed to in the LC-SRC, then it does stay with you!” (I-15).*

### Collaboration

The third theme, collaboration, consisted of collaboration with other students, with student coordinators, and with clinical supervisors. Collaboration ensures patient safety and motivates students, and in this supervised practice creates a valuable patient-centred teaching opportunity for students. Collaboration not only enabled the students to learn to work together, but also helped the students to see the value of sharing knowledge and skills. Collaboration contributed to better outcomes in terms of both patient management and what students learned.

### Collaboration with students

Students who participated in the LC-SRC felt special, being part of a special group. They saw collaboration as both an opportunity (especially the contact with students in higher study years) and a threat (thrilling to work together in a high-stakes situation with other unknown students). This collaboration was different from that of their regular curriculum, where they collaborate with fellow students in the same study year. They considered the latter as inefficient and unnecessary. When preparing for the consultations, the student participants found collaborating with fellow students to be supportive, pleasant, and effective, and especially when they worked with students from other study years, which they considered to be particularly educational and motivating.

*"The collaboration actually went well. You noticed the different phases of the study between the students, but that connected well and thereby we learned. Of course, a fifth-year student knows more compared than a third-year student, who knows more than a first-year student. Therefore, it was very exciting, to be able to exchange our knowledge and still think hard to come up with a diagnosis and how to treat it. We all learned individually; however most of all we learned lots from each other." (I-02).*

The team atmosphere was important. In the interaction with patients and clinical supervisors, the student teams felt they complemented each other and experienced collaboration as a safety net, so that they would not miss important clues or topics during the consultation and discussion with the clinical supervisor. Thereby they felt that they had learned a lot and had progressed further in their development as future doctors.

### Collaboration with student coordinators

The student coordinators play an important role in facilitating other students’ learning and preparation for their consultations. They appreciated that they had to be careful not to intervene too much or too soon when student participants prepared their plans for consultation and treatment.

“I think it is very important not to intervene too soon, you have to let the students make a plan. I really think that is the most educational. But also taking your time, not hastily commenting ‘it’s alright’ or something, but really discussing what the students came up with and why.” (I-08).

Student participants regarded the student coordinators as very approachable and helpful, and liked the fact they were students themselves. The student coordinators considered their role, functioning as a bridge between student participants and clinical supervisors, to be a valuable educational and teaching/coaching experience. Both student participants and student coordinators felt they learned from each other.

### Collaboration with clinical supervisors

Both student participants and coordinators found it an exciting experience to collaborate with the clinical supervisors. Some mentioned that they particularly liked the opportunity to meet and work alongside experienced clinical supervisors. While the supervisors were often busy with clinical tasks, they remained accessible and enthusiastic, willing to answer questions and to coach students. This not only guaranteed patient safety but also provided students with extra teaching because the clinical supervisors often asked them additional questions..

*“Of course, they [clinical supervisors] supervise us closely. After each consultation, we had to call them so they could come by or discuss the case. I think that is a very nice approach. […] you get the confirmation you did well and when necessary you get some additional comments; Nevertheless, you do it all yourself!” (I-18).*

Students perceived the interaction with the clinical supervisors as instructive but also as challenging – they wanted to live up to expectations of the clinical supervisors. Student participants appreciated that the clinical supervisors took time for them and recognized them as individuals. This helped student participants to feel at ease in a new setting, such that they thought that future clinical encounters would be less stressful.

### Framework of learning in an LC-SRC

**Fig. 1:**
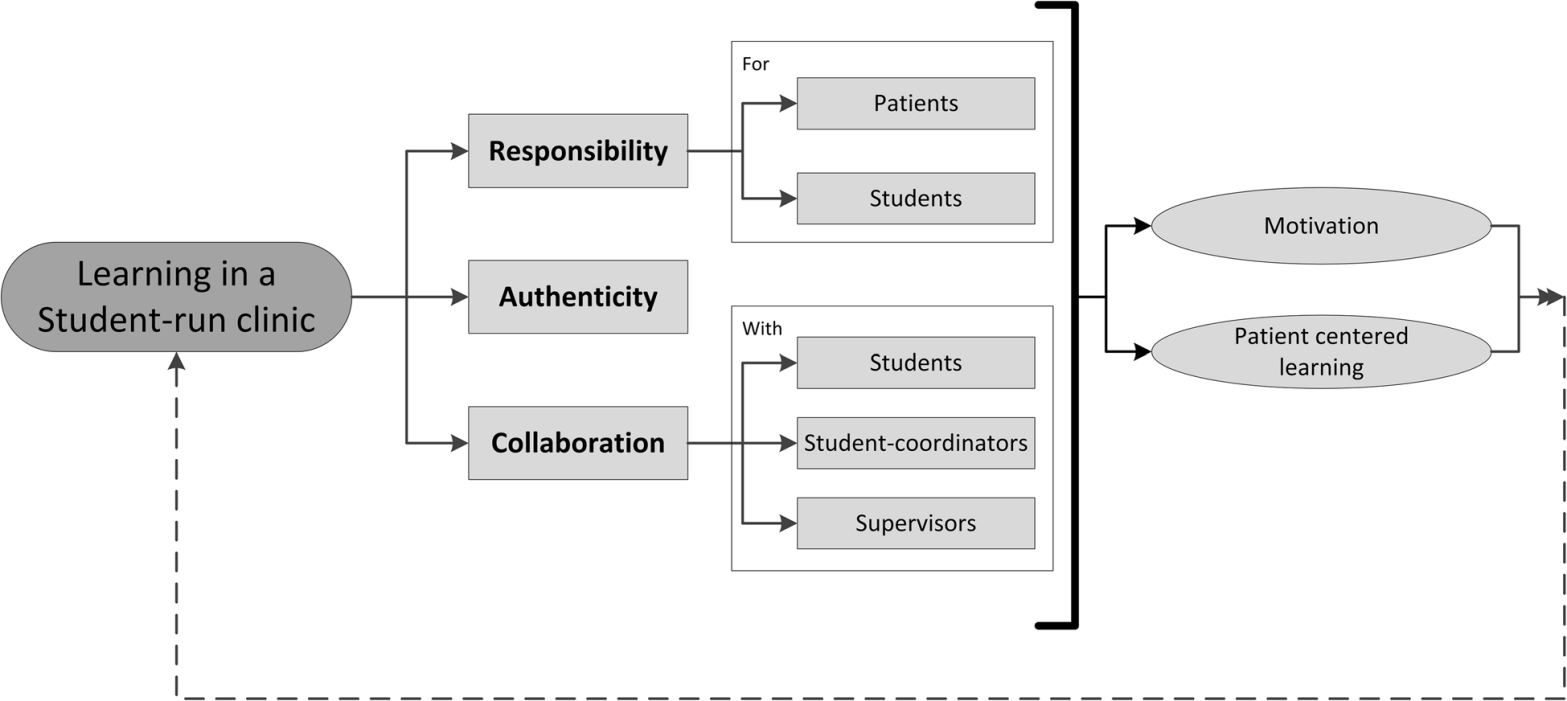
Framework of learning in a student-run clinic (SRC). The three factors, Responsibility, Authenticity, and Collaboration are the main themes underlying student learning in SRCs. These three factors promote student motivation and drive patient-centred learning

Responsibility, authenticity, and collaboration were identified as key individual and interacting components of learning in an LC-SRC (Fig. 1). The learning situation in an LC-SRC is authentic because the student participants have to collaborate with colleagues and have responsibility for real patients, as they would in clinical practice. The student coordinators in turn have responsibility for the participating students, working with the students and clinical supervisors to ensure that patients are treated appropriately. Together, the elements of responsibility, authenticity, and collaboration motivated students and drove patient-centred learning. The students described this motivation as arising from a genuine interest in their patients, which in turn prompted them to learn. In working with real patients, the student participants learned how to deal with uncertainty and in some cases about the long-term management of patients. They also learned from the patient feedback. This learning had nothing to do with cramming up on facts. The students learned in networks, while combining forward and backward reasoning, and transferred this patient-centred learning to their regular curriculum. In this way, LC-SRC participation contributes to education and learning.

## Discussion

In this qualitative semi-structured interview study, we investigated how participation in an LC-SRC contributes to learning and how this learning is influenced. We found responsibility, authenticity, and collaboration to be the main themes underlying student learning in an LC-SRC, and together these themes enhanced motivation and promoted patient-centred learning in both the LC-SRC and the regular curriculum. This clarifies how SRC participation contributes to learning. These results resonate to several concepts or theories on workplace learning that we discuss hereafter.

Learning in the workplace by “supported participation” is the central theme in the experienced-based learning (ExBL) model of Dornan et al. and Billett’s general pedagogy of workplace learning [23, 24]. Our findings are consistent with their findings regarding the importance of authenticity (related to participation) and collaboration (relating to support). However, some important differences appear to exist between these models and our LC-SRC model. The ExBL model seems more passive than our model, especially regarding the balance between support, responsibility, and self-directedness. The ExBL model is primarily focused on support and does not regard self-direction and responsibility as core conditions for experience-based learning [25, 26]. Given the minimal attention for responsibility and self-direction, participation in an ExBL setting does not appear to foster autonomy or engagement relative to participation in the authentic setting of an LC-SRC. Another, minor, difference between the models is the difference in importance attached to collaboration in teams and peer teaching. We found collaboration to be a major component of the LC-SRC learning model. Thus the current ExBL model appears to be incomplete in terms of responsibility and collaboration, so that current theory on practice-based learning may need to be adapted to include these aspects. The ExBL framework itself is closely related to both the Situated Learning theory (SLT) of Lave and Wenger, and Vygotsky’s Zone of Proximal Development (ZPD) [25, 27, 28].

In their SLT, Lave and Wenger described the role of communities of practice [27]. In these communities of practice, students participate in shared activities and social interaction in groups, which is considered key to the formation of a professional identity. Students start at the periphery and evolve as they gain more experience and are finally able to participate as professionals in the desired community of practice [27]. The importance of interactions in teams with students and professionals was pivotal to our model and is consistent with the tenets of the SLT. Although the two models emphasize the importance of teamwork and collaboration, the SLT does not include the element of responsibility. In the SLT, student participation is far more peripheral and does not contribute directly to real patient care.

The ZPD of Vygotsky holds that student learning can be maximized by assigning students tasks that are just within their capabilities if they have assistance and appropriate scaffolding [28, 29]. However, providing too much assistance or structure may hinder learning – students will complete the task, but they will not learn to do the task independently [29]. In our study, this balance between optimal challenge and support was reflected by students commenting that while LC-SRC participation was highly challenging on the one hand, they felt that they were supported by the coordinators and supervisors on the other. Compared with the ZPD, the ExBL and SLT models seem more gradual, less self-directed, more focused on the professional identity and less on mastering skills and acquiring knowledge. Both the findings from our present study and ZPD theory corroborate the previously mentioned issue regarding the lack of attention for responsibility and collaboration in the current ExBL and SLT models and highlight the need to take these issues into account in workplace-based learning.

### Implications for future research

The absence of responsibility as an important factor in most current models and theories of learning in the workplace is noticeable. It would be interesting to explore the potential positive and negative effects of responsibility. Potential positive effects include improved learning outcomes, greater self-confidence, and better readiness for clinical practice. Potential negative effects are stress and uncertainty. Now that we have gained a better understanding of the factors underlying learning in an LC-SRC, we can improve and re-evaluate the LC-SCR concept and possibly apply these learning facilitators to other tasks important for our future doctors, in order to optimize education in patient management and drug safety.

### Strengths and limitations

To the best of our knowledge, this is the first clarification study into the mechanisms underlying learning in an LC-SRC. Our findings have clear links to existing literature and are grounded in learning theories. The main limitation of this study is the single setting design. Moreover, the setting is essential in establishing a rich learning environment, and different settings offer different learning opportunities. Our European LC-SRC is different from many SRC’s in the USA, regarding for example caring for the underserved. Although “what” students learn may be different, we expect our findings regarding “how” they learn to be the same. Another limitation is the (purposeful) selection of eligible interviewees and self-selection bias for participating in the LC-SRC, which may influence the generalizability of our findings. Moreover the voluntary status of this project could influence the generalizability of our findings regarding the identified themes. Making the project a compulsory part of the medical curriculum could hinder the students` feelings of autonomy. Nevertheless the implementation and arrangement of responsibilities would presumably have more impact on the generalizability of the themes responsibility and authenticity than the voluntary/compulsory status of the project.

## Conclusions

This study showed that SRC participation contributes to learning by offering students a rich learning environment with responsibility, authenticity, and collaboration as the three main underlying themes. Learning in an LC-SRC is highly dependent on students’ feelings of responsibility for a real authentic task and is stimulated by extensive collaboration with fellow students and supervising doctors. Thereby, participation in an LC-SRC, with involvement in real patient care, offers extensive learning opportunities and is highly motivating for students.

## Additional file

Additional files 1: Topic List: Topic list and list of (examples of) questions used during the interviews. (DOCX 19 kb)

## Abbreviation

COREQ: Consolidated criteria for Reporting Qualitative studies
ExBL: Experienced-based learning
LC-SRC: Learner-centred student-run clinic.
SLT: Situated Learning theory
SRCs: Student-run clinics
ZPD: Zone of Proximal Development

## References

[1] Schutte T, Tichelaar J, Dekker RS, van Agtmael MA, de Vries TP, Richir MC (2015). Learning in student-run clinics: a systematic review. Med Educ.

[2] Tong ST, Phillips RL, Berman R (2012). Is exposure to a student-run clinic associated with future primary care practice?. Fam Med.

[3] Smith SD, Johnson ML, Rodriguez N, Moutier C, Beck E (2012). Medical student perceptions of the educational value of a student-run free clinic. Fam Med.

[4] Smith S, Rr T, Cruz M, Griggs R, Moscato B, Ferrara A (2014). Presence and characteristics of student-run free clinics in medical schools. JAMA.

[5] Stephens L, Bouvier N, Thomas D, Meah Y (2015). Voluntary participation in a medical student-organized Clinic for Uninsured Patients Significantly Augments the formal curriculum in teaching underrepresented Core competencies. Journal of Student-Run Clinics.

[6] Sheu L, O'Brien B, O'Sullivan PS, Kwong A, Lai CJ (2013). Systems-based practice learning opportunities in student-run clinics: a qualitative analysis of student experiences. Acad Med.

[7] Davenport BA (2000). Witnessing and the medical gaze: how medical students learn to see at a free clinic for the homeless. Med Anthropol Q.

[8] Batra P, Chertok JS, Fisher CE, Manseau MW, Manuelli VN, Spears J (2009). The Columbia-Harlem homeless medical partnership: a new model for learning in the service of those in medical need. J Urban Health.

[9] Lie DA, Forest CP, Walsh A, Banzali Y, Lohenry K (2016). What and how do students learn in an interprofessional student-run clinic? An educational framework for team-based care. Med Educ Online.

[10] Chen HC, Sheu L, O'Sullivan P, Ten Cate O, Teherani A (2014). Legitimate workplace roles and activities for early learners. Med Educ.

[11] van der Zwet J, Hanssen VG, Zwietering PJ, Muijtjens AM, van der Vleuten CP, Metsemakers JF, Scherpbier AJ (2010). Workplace learning in general practice: supervision, patient mix and independence emerge from the black box once again. Med Teach.

[12] Tichelaar J, van Kan C, van Unen RJ, Schneider AJ, van Agtmael MA, de Vries TP, Richir MC (2015). The effect of different levels of realism of context learning on the prescribing competencies of medical students during the clinical clerkship in internal medicine: an exploratory study. Eur J Clin Pharmacol.

[13] Celebi N, Kirchhoff K, Lammerding-Koppel M, Riessen R, Weyrich P (2010). Medical clerkships do not reduce common prescription errors among medical students. Naunyn Schmiedeberg's Arch Pharmacol.

[14] Cook DA, Bordage G, Schmidt HG (2008). Description, justification and clarification: a framework for classifying the purposes of research in medical education. Med Educ.

[15] Dekker RS, Schutte T, Tichelaar J, Thijs A, van Agtmael MA, de Vries TP, Richir MC (2015). A novel approach to teaching pharmacotherapeutics--feasibility of the learner-centered student-run clinic. Eur J Clin Pharmacol.

[16] Schutte Tim, Tichelaar Jelle, van Agtmael Michiel (2015). Learning to prescribe in a student-run clinic. Medical Teacher.

[17] Schutte T, Tichelaar J, Reumerman MO, van Eekeren R, Rolfes L, van Puijenbroek EP, Richir MC, van Agtmael MA (2017). Feasibility and educational value of a student-run pharmacovigilance Programme: a prospective cohort study. Drug Saf.

[18] Schutte T, Prince K, Richir M, Donker E, van Gastel L, Bastiaans F, de Vries H, Tichelaar J, van Agtmael M (2018). Opportunities for students to prescribe: an evaluation of 185 consultations in the student-run cardiovascular risk management Programme. Basic Clin Pharmacol Toxicol.

[19] Glaser BG. Emergence vs forcing: basics of grounded theory analysis. Mill Valley, Caliornia, USA, Sociology press; 1992.

[20] Glaser BG, Strauss AL. The discovery of grounded theory: strategies for qualitative research. Chicago: Aldine; 1967.

[21] Westerman M, Teunissen PW, van der Vleuten CP, Scherpbier AJ, Siegert CE, van der Lee N, Scheele F (2010). Understanding the transition from resident to attending physician: a transdisciplinary, qualitative study. Acad Med.

[22] Tong A, Sainsbury P, Craig J (2007). Consolidated criteria for reporting qualitative research (COREQ): a 32-item checklist for interviews and focus groups. Int J Qual Health Care.

[23] Dornan T, Boshuizen H, King N, Scherpbier A (2007). Experience-based learning: a model linking the processes and outcomes of medical students' workplace learning. Med Educ.

[24] Billett S (2002). Toward a workplace pedagogy: guidance, participation, and engagement. Adult Educ Q.

[25] Yardley S, Teunissen PW, Dornan T (2012). Experiential learning: AMEE guide no. 63. Med Teach.

[26] Dornan T, Hadfield J, Brown M, Boshuizen H, Scherpbier A (2005). How can medical students learn in a self-directed way in the clinical environment? Design-based research. Med Educ.

[27] Lave J, Wenger E (1991). Situated learning: legitimate peripheral participation.

[28] Vygotsky LS: Mind and society. Cambridge, Massachusetts (USA): Harvard University press written in 1930, 1978.

[29] Wass R, Golding C (2014). Sharpening a tool for teaching: the zone of proximal development. Teach High Educ.

