# Supplementary material for: Clarifying learning experiences in student-run clinics: a qualitative study
Source: BMC Med Educ. 2018 Oct 26;18:244. doi: 10.1186/s12909-018-1352-6 (PMC6204044; doi:10.1186/s12909-018-1352-6)
Supplement: Supplementary file 1 — Topic List: Topic list and list of (examples of) questions used during the interviews. (DOCX 19 kb) [file 12909_2018_1352_MOESM1_ESM.docx]

Topic List

**Clarifying learning experiences in student-run clinics:  a qualitative study**

Tim Schutte^1,2^, Jelle Tichelaar^1,2^, Erik Donker^1,2^, Milan C. Richir^1,2^, Michiel Westerman^3,4^, Michiel A. van Agtmael^1,2^

1 Amsterdam UMC, Vrije Universiteit Amsterdam, department of Internal Medicine, pharmacotherapy section

2 RECIPE (Research & Expertise Center In Pharmacotherapy Education), Amsterdam, the Netherlands

3 Amsterdam UMC, Vrije Universiteit Amsterdam, Department of Internal Medicine VU University Medical Center, Amsterdam, the Netherlands

4 Amsterdam UMC, Vrije Universiteit Amsterdam/VU University Medical Center School of Medical Sciences, Amsterdam, the Netherlands

**Running Head:** Clarifying learning in student-run clinics

**Corresponding author:**

Tim Schutte, [t.schutte@vumc.nl](mailto:t.schutte@vumc.nl) ORCID: 0000-0002-4096-0917

Amsterdam UMC, Vrije Universiteit Amsterdam,

Department of Internal Medicine, Pharmacotherapy Section,

De Boelelaan 1118 1081 HZ, Amsterdam, The Netherlands

Phone +31 20 4448090

**Topics and Questions (examples)**

| Topic | Question (examples) |
| --- | --- |
| Interest and motivation | - How did you get interested and involved in the LC-SRC? - What did you expect by participating? |
| Preparation | - In which way did you prepare for a consultation? - Was this preparation different when you gained experience? |
| Interaction with supervisors and supervising students | - How did you perceive the interaction between other (coordinating) students and supervisors? |
| Learning activities and patient interaction | - What was your task within the LC-SRC? - Can you tell us something about your patient encounters? |
| Reflection on participation | - How would you reflect on your participation in the LC-SRC? - Can you tell us something about how you learned in the LC-SRC? - How did you experience the difference between the LC-SRC and the regular curriculum? |
| Facilitators and barriers | - Which facilitators and barriers did you encounter while participating in the LC-SRC - Did you encounter problems during your participation? - Which factors are essential to make the LC-SRC a success? |
